# E2F2 Reprograms Macrophage Function By Modulating Material and Energy Metabolism in the Progression of Metabolic Dysfunction‐Associated Steatohepatitis

**DOI:** 10.1002/advs.202410880

**Published:** 2024-10-28

**Authors:** Zheng Liu, Hao Wang, Yuan Liang, Mu Liu, Qiyuan Huang, Mingming Wang, Jinren Zhou, Qingfa Bu, Haoming Zhou, Ling Lu

**Affiliations:** ^1^ Department of General Surgery the First Affiliated Hospital of Nanjing Medical University Nanjing 210029 China; ^2^ Department of Liver Surgery State Key Laboratory of Complex Severe and Rare Diseases Peking Union Medical College Hospital Chinese Academy of Medical Sciences and Peking Union Medical College Beijing 100730 China; ^3^ Hepatobiliary Center The First Affiliated Hospital of Nanjing Medical University & Research Unit of Liver Transplantation and Transplant Immunology Chinese Academy of Medical Sciences Nanjing 210029 China; ^4^ School of Biological Science & Medical Engineering Southeast University Nanjing 210096 China; ^5^ Affiliated Hospital of Xuzhou Medical University Xuzhou 220005 China

**Keywords:** amino acid transportation, glycolysis, macrophage, metabolic dysfunction‐associated steatohepatitis, slc7a5

## Abstract

Macrophages are essential for the development of steatosis, hepatic inflammation, and fibrosis in metabolic dysfunction‐associated steatohepatitis(MASH). However, the roles of macrophage E2F2 in the progression of MASH have not been elucidated. This study reveals that the expression of macrophage E2F2 is dramatically downregulated in MASH livers from mice and humans, and that this expression is adversely correlated with the severity of the disease. Myeloid‐specific E2F2 depletion aggravates intrahepatic inflammation, hepatic stellate cell activation, and hepatocyte lipid accumulation during MASH progression. Mechanistically, E2F2 can inhibit the SLC7A5 transcription directly. E2F2 deficiency upregulates the expression of SLC7A5 to mediate amino acids flux, resulting in enhanced glycolysis, impaired mitochondrial function, and increased macrophages proinflammatory response in a Leu‐mTORC1‐dependent manner. Moreover, bioinformatics analysis and CUT &Tag assay identify the direct binding of Nrf2 to E2F2 promoter to promote its transcription and nuclear translocation. Genetic or pharmacological activation of Nrf2 effectively activates E2F2 to attenuate the MASH progression. Finally, patients treated with CDK4/6 inhibitors demonstrate reduced E2F2 activity but increased SLC7A5 activity in PBMCs. These findings indicated macrophage E2F2 suppresses MASH progression by reprogramming amino acid metabolism via SLC7A5‐ Leu‐mTORC1 signaling pathway. Activating E2F2 holds promise as a therapeutic strategy for MASH.

## Graphical abstract

E2F2 deficiency regulated amino acids metabolism to promote glycolysis and mitochondrial dysfunction, and in turn enhance macrophage proinflammatory response. Finally resulting in the lipid accumulation of hepatocytes and the activation of hepatic stellate cells (HSCs) aggravated metabolic dysfunction‐associated steatohepatitis development.

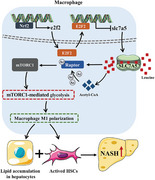

## 1 Introduction

Due to its close relationship to obesity, metabolic dysfunction‐associated steatotic liver disease(MASLD), has become a major worldwide health problem.^[^ ^1^, ^2^ ^]^ This disorder includes a variety of liver diseases, ranging from simple steatosis (also known as metabolic dysfunction‐associated steatotic liver, MASL) to metabolic dysfunction‐associated steatohepatitis(MASH), which can lead to cirrhosis and hepatocellular carcinoma (HCC).^[^ ^3^, ^4^, ^5^ ^]^ Numerous simultaneous intrahepatic and extrahepatic events have been identified as part of the large‐scale investigation into the pathophysiology of MASLD and MASH. It has been shown that inflammation associated with macrophages and lipid metabolism are important factors in controlling the course of MASH.^[^ ^6^, ^7^, ^8^ ^]^ Furthermore, research has demonstrated that along the course of MASH, hepatic hyperglycemia, bile acid toxicity, and hepatic stellate cell activation all contribute to increasing liver damage.^[^ ^9^ ^]^ Growing evidence supports the view that innate immunity is one of the most essential facets of MASLD.^[^ ^10^, ^11^ ^]^

Specifically, it is thought that macrophages are important for the development of fibrosis, inflammation, and steatosis in MASH.^[^ ^12^ ^]^ Macrophage differentiation, polarization, mobilization, and antitumor response are regulated by metabolism.^[^ ^13^ ^]^ Macrophage activity depends on metabolic processes such as fatty acid production, glycolysis, the tricarboxylic acid (TCA) cycle, the pentose phosphate pathway, and amino acid (AA) metabolism.^[^ ^14^ ^]^ In order to satisfy the demands of their engagement in diverse immunological activities, activated macrophages must closely regulate their metabolic programs, as immune responses are energy‐demanding biosynthetic processes.^[^ ^15^, ^16^ ^]^ In LPS‐activated macrophages, enhanced glycolysis‐mediated glucose uptake produces sufficient ATP and biosynthetic intermediates to maintain effector functions.^[^ ^14^, ^17^, ^18^ ^]^ In addition to glycolysis, the uptake of amino acids is necessary for protein synthesis to contribute to cell proliferation and cytokine production during macrophage activation.^[^ ^14^ ^]^ Additionally, amino acids are broken down into metabolic intermediates through catabolic pathways, contributing to numerous metabolic processes.^[^ ^19^ ^]^ Recently, studies have reported that AAs, like leucine and arginine, directly bind to sensing molecules to activate the mTOR complex 1 (mTORC1).^[^ ^20^, ^21^ ^]^ Yet, the specific mechanisms of activated macrophages to regulate metabolic pathways in MASH remain unelucidated and require better characterization. Effective therapy solutions require an understanding of the molecular processes underlying MASH.

E2F2, a transcription factor, is a significant regulator of proliferation, differentiation, and apoptosis. Studies have shown that E2F2 protects against oxidative stress by promoting the NRF2‐ROS signaling axis and is closely associated with the IKK/NF‐κB signaling pathway.^[^ ^22^, ^23^, ^24^ ^]^ Furthermore, E2F2 has been identified as playing a pivotal role in regulating key metabolic enzymes involved in MASLD‐related hepatocarcinogenesis.^[^ ^25^ ^]^ The lack of E2F2 develops late‐onset autoimmune features. Lacking E2F2, T cells proliferate more in response to TCRs and have a lower activation threshold, which causes autoreactive effector/memory T cells to accumulate.^[^ ^26^ ^]^ It is still unclear how E2F2 affects macrophage function, specifically in controlling hepatic inflammation, fibrosis, and dysregulated lipid metabolism in MASH.

In this work, we explored the function of E2F2 in macrophages during the development of MASH using macrophage‐specific E2F2 knockout (E2F2^M‐KO^) and NRF2 knockout (NRF2^M‐KO^) mice.

## 2 Results

### 2.1 E2F2 Levels are Downregulated in the Macrophages of Mouse and Human MASH Livers, and Negatively Correlated with Disease Severity

**Figure 1:**
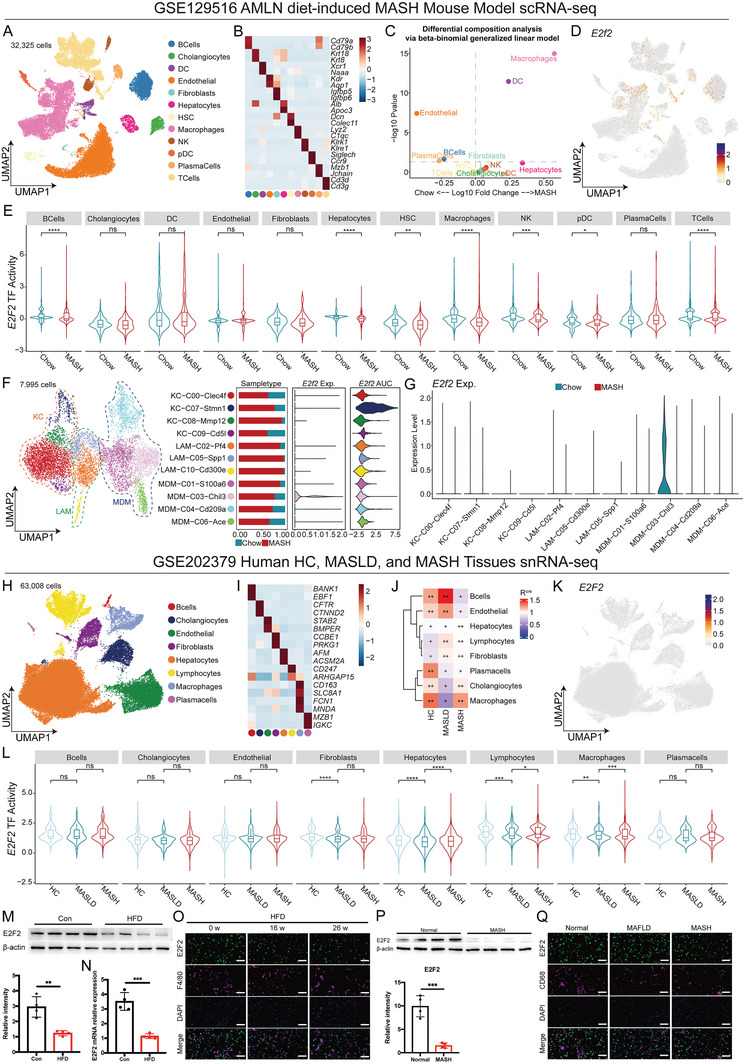
E2F2 levels are down‐regulated in the macrophages of mouse and human MASH livers, and negatively correlated with disease severity. A) A Uniform Manifold Approximation and Projection (UMAP) shows 32, 325 cells from 3 MASH mouse samples and 3 chow mouse samples, colored by major cell types. B) Heatmap showing the mean expression of top2 markers of each cell type. C) Dot plot showing the enrichment of major cell types via beta‐binomial generalized linear model. colors represent the differential cell types. D) UMAP plot showing the RNA expression of E2f2. E) Box plots showing the predicted transcriptional level of E2f2 in the major cell types between the Chow and MASH tissues. Colors represent the sample types. F) UMAP plot showing the specific clusters of macrophages (left). Bar plots showing the ratio of sample type in each cluster, violin plots showing the RNA expression of Ef2 in each cluster, and violin plots showing the transcriptional level of E2f2 (from left to right). G) Violin plots showing the RNA expression of E2f2 between Chow and MASH tissues in each cluster. H). UMAP plot showing the major cell types in the human healthy control, MASLD, and MASH tissues from GSE202379. I) Heatmap showing the mean expression of top 2 markers of each cell type. J) Heatmap showing the result of the R^o/e^. K) UMAP plot showing the RNA expression of E2F2. L) Box plots showing the transcriptional level of E2F2 among disease stages in each cell type. M,N) E2F2 Protein and mRNA levels in hepatic macrophages isolated from WT mice livers. WT mice were fed the HFD, or control diet. O) Representative IF images showing E2F2 and F4/80 (green and red) in liver sections from WT mice fed the HFD, or control diet. n = 6/group; P) E2F2 Protein levels in hepatic macrophages isolated from human subjects without steatosis or with MASH livers. Q) Representative IF images showing E2F2 and CD68 (green and red) in liver sections from human subjects without steatosis, with simple steatosis, and with MASH. Data were presented as mean± SEM; Scale bars, 100µm; *p ≤ 0.05, **p ≤ 0.01, ***p ≤ 0.001.

To investigate the involvement of macrophage E2F2 in MASH, we re‐analyzed the scRNA‐seq data from the livers of diet‐induced mouse MASH models (GSE129516). Twelve main cell types expressing known marker genes were identified including B cells (Cd79a, Cd79b), cholangiocytes (Krt18, Krt8), dendritic cells (DC) (Xcr1, Naaa), endothelial cells (Kdr, Aqp1), fibroblasts (Igfbp5, Igfbp6), hepatocytes (Alb, Apoc3), hepatic stellate cells (HSCs) (Dcn, Colec11), hepatocytes (Alb, Fabp1), macrophages (Lyz2, C1qc), natural killer cells (NK) (Klrk1, Klre1), plasmacytoid dendritic cells (pDC) (Siglech, Ccr9), plasma cells (Mzb1, Jchain), and T cells (Cd3d, Cd3e) (**Figure** **1**A,B). Further, differential composition analysis via beta‐binomial generalized linear model revealed that macrophages were significantly enriched in MASH tissues, which might play a crucial role in the MASH progression (Figure 1C). The expression of E2f2 was further investigated, and it was found that its RNA expression level was low, and it was mainly expressed in macrophages, B cells, DC cells, and T cells (Figure 1D). Using the pyscenic algorithm, the transcriptional activity of E2F2, a transcription factor belonging to the E2F family, was further examined. The results indicated that E2F2 exhibited higher transcriptional activity in chow‐fed tissues compared to MASH tissues, particularly in macrophages(Figure 1E). We further re‐clustered macrophages into 11 clusters, including four Kupffer cells (KC) (KC‐C00‐Clec4f, KC‐C07‐Stmn1, KC‐C08‐Mmp12, and KC‐C09‐Cd5l), three lipid‐associated macrophages (LAM) (LAM‐C02‐Pf4, LAM‐C05‐Spp1, and LAM‐C10‐Cd300e), and four monocyte‐derived macrophages (MDM) (MDM‐C01‐S100a6, MDM‐C03‐Chil3, MDM‐C04‐Cd209a, and MDM‐C06‐Ace) (Figure 1F). We observed that the RNA expression of E2f2 was significantly expressed in the MDM‐C03‐Chil3. In addition, the MDM‐C03‐Chil3 also had strong transcription factor activity of E2f2 (Figure 1F). Notably, the higher E2f2 RNA expression in the MDM‐C03‐Chil3 was in the chow tissues compared with the MASH tissues (Figure 1G). To confirm this finding, we explored E2F2 expression in hepatic macrophages isolated from a methionine‐ and choline‐deficient diet (MCD) or a high‐fat diet (HFD) induced mouse MASH models. Consistently, both E2F2 mRNA and protein levels were significantly decreased in hepatic macrophages (Figure 1M,N and Figure S1A,B, Supporting Information) from dietary‐induced MASH mouse models compared with those fed with normal chow but not in other kinds of cells, including HSCs, ductular cells, endothelial cells, hepatocytes (Figure S1C, Supporting Information) and neutrophils (Figure S1D, Supporting Information). Furthermore, decreased staining of E2F2 was observed in F4/80 positive macrophages as well, and interestingly, macrophage E2F2 expression levels were negatively correlated with MASH progression in the liver of HFD‐fed mice (Figure 1O). Contrastingly, no significant difference in E2F2 staining in hepatocytes (Figure S1E, Supporting Information), HSCs (Figure S1F, Supporting Information), and ductular cells (Figure S1G, Supporting Information).

Meanwhile, to test the clinical significance of hepatic macrophage E2F2 expression, we also re‐analyzed the human snRNA‐seq data from healthy control, MASLD, and MASH liver tissues (GSE202379). Eight main cell types expressing known marker genes were identified including B cells (BANK1, EBF1), cholangiocytes (CFTR, CTNND2), endothelial cells (STAB2, BMPER), fibroblasts (CCBE1, PRKG1), hepatocytes (AFM, ACSM2A), lymphocytes (CD247, ARHGAP15), macrophages (CD163, SLC8A1, FCN1, and MNDA), and plasma cells (MZB1, IGKC) (Figure 1H,I). Further, macrophages were significantly enriched in MASH tissues via the Ro/e algorithm (Figure 1J). The RNA expression of E2f2 was low (Figure 1K), however, the transcriptional activity of E2F2 showed a step‐down trend with disease progression (Figure 1L).

Next, we collected liver tissues from human subjects without steatosis, with simple steatosis, and with MASH (Figure S1I, Supporting Information). E2F2 levels were considerably lower in the livers of people with simple steatosis or MASH compared to those without steatosis, which is consistent with our findings in mice. Furthermore, E2F2 expression was markedly reduced in hepatic macrophages of the MASH group compared to the simple steatosis group (Figure 1P,Q), whereas no significant differences were observed in other cell types (Figure S1H, Supporting Information), including hepatocytes (Figure S1J, Supporting Information), HSCs (Figure S1K, Supporting Information), and ductular cells (Figure S1L, Supporting Information). Overall, these findings showed that throughout the transition from a normal liver to MASLD and MASH, E2F2 is down‐regulated in hepatic macrophages in both human and mouse livers.

### 2.2 Myeloid‐Specific E2F2 Deficiency Aggravates the MASH Progression

**Figure 2:**
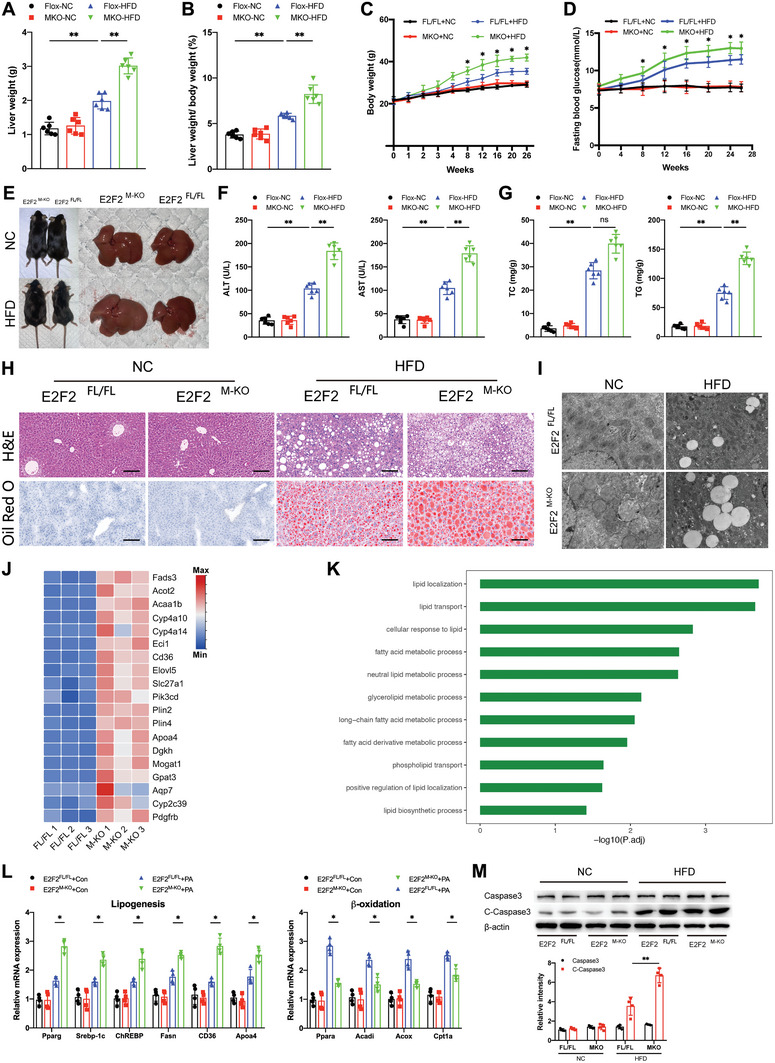
Myeloid‐specific E2F2 deficiency aggravates the MASH progression. A–D) Liver weights (A), ratios of liver weight to body weight (B), body weight (C), and fasting blood glucose (D) of E2F2^M‐KO^ mice and their corresponding E2F2^FL/FL^ control after NC or HFD consumption (n = 6/group). E) Representative photographs of liver tissues from E2F2^M‐KO^ and E2F2^FL/FL^ mice fed with NC or HFD (n = 6/group). F) Serum ALT and AST concentrations in the indicated groups of mice (n = 6/group). G) Hepatic TG and TC content of mice in the indicated groups (n = 6/group). H,I) H&E, Oil Red O (H), and TEM (I) in liver sections from E2F2^M‐KO^ and E2F2^FL/FL^ mice fed with NC or HFD. (n = 6/group). J,K) Heatmap (J) and GO (K) reveal the signature genes and up‐regulated signaling pathways related to lipid metabolism in the liver samples of E2F2^M‐KO^ mice compared with E2F2^FL/FL^ mice. E2F2^M‐KO^ and E2F2^FL/FL^ mice were fed with HFD. (n = 3/group). L) The mRNA levels of lipogenesis genes (Pparg, Srebp‐1c, ChREBP, CD36, Apoa4 and Fasn) and β‐oxidation genes (Ppara, Acadi, Acox, and Cpt1a) in primary hepatocytes co‐cultured with BMDMs from E2F2^M‐KO^ and E2F2^FL/FL^ mice in palmitic acid (PA) medium for 24 h. M) Representative Western blot of Caspase3, cleaved Caspase3 (C‐Caspase3) and β‐catin in liver tissues from E2F2^M‐KO^ and E2F2^FL/FL^ mice fed with NC or HFD. n = 6/group. Data were presented as mean± SEM; Scale bars, 100µm; *p ≤ 0.05, **p ≤ 0.01, ***p ≤ 0.001.

In order to examine the function of macrophage E2F2 in the advancement of MASH and the corresponding macrophage infiltration, we employed the Cre‐LoxP system to generate myeloid‐specific E2F2‐deficient mice (E2F2^M‐KO^). In these E2F2^M‐KO^ mice, E2F2 expression was significantly deficient in BMDMs, although similar in other kinds of cells, such as neutrophils, KCs, and hepatocytes (Figure S2A, Supporting Information). E2F2^M‐KO^ mice and E2F2^FL/FL^ controls were fed either a normal chow diet, MCD for 6 weeks, or HFD for 26 weeks. In comparison to the E2F2^FL/FL^ controls, the E2F2^M‐KO^ group displayed higher body weight, fasting blood glucose levels, liver weights, and liver‐to‐body weight ratios after 26 weeks on the HFD (**Figure** **2**A–E). Consequently, serum levels of alanine aminotransferase (ALT) and aspartate aminotransferase (AST), as well as hepatic triglyceride (TG) and total cholesterol (TC) concentrations, were significantly elevated in E2F2^M‐KO^ mice compared to E2F2^FL/FL^ controls after HFD feeding(Figure 2F,G). Additionally, as shown by H&E, Oil Red O staining (Figure 2H; Figure S2B, Supporting Information), and TEM (Figure 2G; Figure S2C, Supporting Information), mice fed HFD and MCD had more severe steatosis in their livers than E2F2^FL/FL^ mice on the same diet. Other members of E2F family, including E2F1, E2F3, E2F4, and E2F5 were observed no obvious difference in hepatic macrophages isolated from E2F2^M‐KO^ mice fed with HFD compared to normal chow(NC)choline‐deficient diet (Figure S2D, Supporting Information). GO analysis showed that pathways related to lipid localization, lipid transport, cellular response to lipid, and fatty acid metabolic processes were enriched in the liver of E2F2^M‐KO^ mice, and the heatmap demonstrated the up‐regulated lipid metabolism genes in E2F2^M‐KO^ mice (Figure 2J,K). Quantitative real‐time PCR findings showed that in primary hepatocytes, the depletion of macrophage E2F2 increased the expression of lipogenic genes while decreasing the expression of β‐oxidation‐regulating genes (Figure 2L). Furthermore, macrophage E2F2 depletion aggravated hepatocellular apoptosis by examined cleaved Caspase3 protein levels in the liver tissues from four groups (Figure 2M).

To gain further insight into steatohepatitis, we assessed hepatic inflammation levels. Immunofluorescence staining for F4/80 and CD11b revealed a greater infiltration of macrophages and neutrophils in the livers of E2F2^M‐KO^ mice compared to E2F2^FL/FL^ controls fed either HFD or MCD diet(Figure S2E, Supporting Information). We further confirmed the up‐regulated genes and signaling pathways related to inflammation in the liver sample of HFD‐fed E2F2^M‐KO^ mice by unbiased RNA sequencing (Figure S2E–G, Supporting Information). Comparing the liver tissues of HFD‐fed E2F2^M‐KO^ mice to those of their HFD‐fed E2F2^FL/FL^ counterparts, quantitative real‐time PCR analysis showed significantly higher gene expression levels of pro‐inflammatory cytokines, such as Tnfα, Il6, Il1β, and Cxcl10 (Figure S2H, Supporting Information). To sum up, our findings verify that the decrease of macrophage E2F2 leads to the infiltration of inflammatory cells in the liver and causes hepatocyte lipid buildup, which worsens the development of steatohepatitis in mice.

### 2.3 E2F2 Deficiency Reprograms Amino Acid Metabolism in Macrophages by Upregulating SLC7A5‐Mediated Leucine Transportation

**Figure 3:**
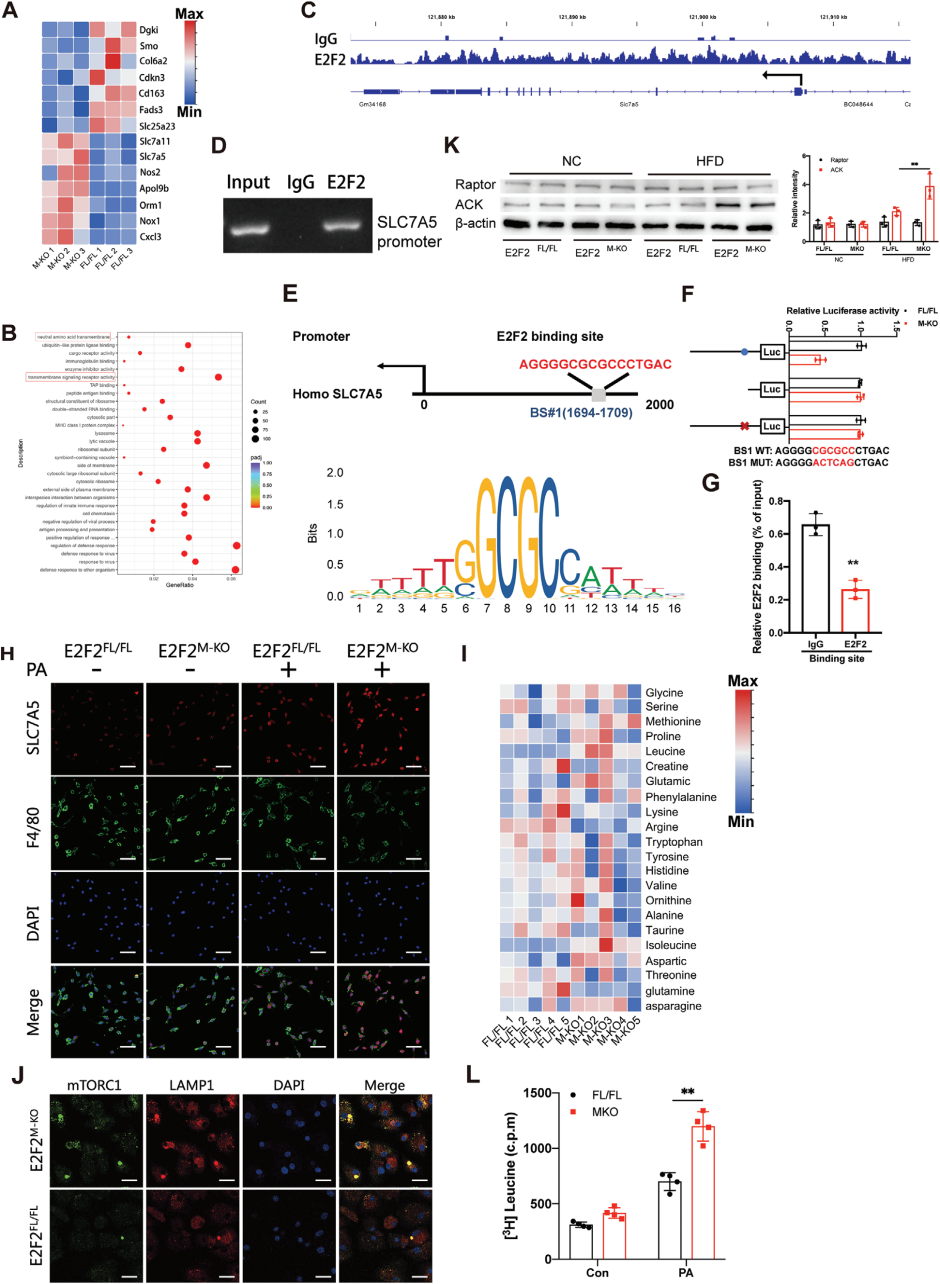
E2F2 deficiency reprograms amino acid metabolism in macrophages by upregulating SLC7A5‐mediated leucine transportation. BMDMs from E2F2^M‐KO^ and E2F2^FL/FL^ mice co‐cultured with primary hepatocytes with or without palmitic acid (PA) medium treated for 24 h. A,B) Heatmap (A) and GO (B) reveal the signature genes and up‐regulated signaling pathways in BMDMs. n = 3/group. C) Representative Integrative Genomics Viewer (IGV) overview of CUT&Tag signals of SLC7A5 gene loci bound by E2F2. n = 6/group. D) ChIP‐PCR analysis of Nrf2 binding to the SLC7A5 promoter. n = 6/group. E) Putative E2F2‐ binding site (BS) within the genomic sequence adjacent to the transcription start site of SLC7A5 gene. F) Luciferase activities of SLC7A5 promoter reporter vectors in THP‐1 cells. Red characters in the binding regions suggest the putative or mutated E2F2‐binding sequences. H) BMDMs were stained with SLC7A5 (red), F4/80 (green), and DAPI (blue). Scale bars, 100µm. I) Heatmap analysis of amino acid metabolism in BMDMs. n = 5/group. J) Representative IF images showing mTORC1 and LAMP1 (green and red) in BMDMs. n = 6/group. K) Western blot analysis of total Raptor, acetylation of Raptor and E2F2 in hepatic macrophages isolated from E2F2^M‐KO^ and E2F2^FL/FL^ mice fed with HFD. n = 6/group. L) Uptake of ^3^H‐leucine in BMDMs. Data were presented as mean± SEM; Scale bars, 100µm; *p ≤ 0.05, **p ≤ 0.01, ***p ≤ 0.001.

RNA‐sequencing study was carried out on bone marrow‐derived macrophages (BMDMs) from E2F2M‐KO and E2F2FL/FL mice in order to examine the molecular processes underlying the function of macrophage E2F2 in MASH. For a duration of 24 hours, these BMDMs were co‐cultured in PA media with primary hepatocytes extracted from WT mice(*n* = 3). In BMDMs lacking E2F2, a significant up‐regulation of SLC7A5 and SLC7A11 gene expression was observed(**Figure** **3**A). Additionally, GO analysis suggested that the neutral amino acid transmembrane transport pathway, along with transmembrane signaling receptor activity, was probably involved in the regulation of E2F2 (Figure 3B). In these E2F2^M‐KO^ mice, SLC7A5 expression was significantly increased in hepatic macrophage, although SLC7A11 expression is similar to E2F2^FL/FL^ mice (Figure S3A). Thus, we hypothesized that E2F2 regulated the transcription of SLC7A5. A potential connection between E2F2 and the SLC7A5 promoter is suggested by the CUT&Tag experiment, which also identified distinct peaks with genomic distribution patterns localized in the promoter regions of SLC7A5 (Figure 3C). After that, we created PCR primers to find the E2F2/TCF DNA‐binding site in the SLC7A5 promoter, and we were able to confirm that E2F2 was present at this area of the promoter (Figure 3D). Using JASPAR, potential E2F2‐binding sites (BS) within the SLC7A5 promoter were predicted, revealing a putative BS1 within the genomic sequence (Figure 3E). Luciferase assays combined with site‐directed mutagenesis and deletion showed that BS1 in the SLC7A5 promoter significantly increased E2F2‐mediated promoter activity in THP‐1 cells (Figure 3F). Moreover, ChIP experiments showed that E2F2 was selectively attracted to THP‐1 cells' BS1 promoter region (Figure 3G). Meanwhile, enhanced SLC7A5 activation was found in liver sections of E2F2^M‐KO^ mice fed with HFD (Figure S3B) and BMDMs from E2F2^M‐KO^ mice co‐cultured with primary hepatocytes in PA medium for 24 h by IF analysis (Figure 3H). In line with these observations, the expression of SLC7A5 in hepatic macrophages was significantly elevated in individuals with simple steatosis or MASH compared to those with non‐steatotic livers. Additionally, subjects with MASH exhibited markedly higher levels of hepatic macrophage SLC7A5 expression than those with simple steatosis (Figure S3C, Supporting Information). Large neutral amino acids (LNAAs), which include essential amino acids (EAAs) like leucine, are transported by SLC7A5. This transporter affects a number of metabolic processes and is essential for the absorption of amino acids by cells.^[^ ^27^ ^]^ Notably, the SLC7A5‐mediated amino acid (AA) influx appears to regulate immune cell activation by modulating the mTORC1‐P70S6K signaling pathway.^[^ ^28^, ^29^ ^]^ A targeted AAs metabolism test was performed on BMDMs from E2F2^M‐KO^ mice and E2F2^FL/FL^ mice co‐cultured with primary hepatocytes obtained from WT mice in PA medium for 24 h in order to ascertain if E2F2 deficiency reprograms macrophage function by up‐regulated SLC7A5‐mediated influx of AAs (*n* = 5). The data confirmed that the levels of SLC7A5‐transported AAs, especially leucine (Leu) and isoleucine were clearly higher (Figure 3I) and phosphorylation of P70S6 kinase (P‐P70S6K) was dramatically increased measured by Western blot and IF staining (Figure S3D,F, Supporting Information)in BMDMs from E2F2^M‐KO^ mice compared to BMDMs from E2F2^FL/FL^ mice. In vivo, IF staining showed enhanced P‐P70S6K activation in liver sections of E2F2^M‐KO^ mice fed with HFD (Figure S3E, Supporting Information).

The conversion of Leu to acetyl‐coenzyme A (AcCoA) favorably controls mTORC1 via Raptor acetylation, and Leu has been linked to mTORC1 activation among AAs. The results showed that in BMDMs from E2F2M‐KO mice, mTORC1 localized in puncta‐like structures and co‐localized with LAMP1‐positive vesicles (late endosomes/lysosomes). However, there was a notable reduction in the redistribution of mTORC1 onto lysosomes in these E2F2‐deficient macrophages(Figure 3J). Hepatic macrophages isolated from E2F2^M‐KO^ mice with HFD showed significantly increased levels of acetylated Raptor (Figure 3K). The important function of E2F2 in regulating SLC7A5 transcription was further shown by the increased inflow of 3H‐leucine in BMDMs deficient in E2F2 (Figure 3L). Furthermore, we used LV‐E2F2 to knockdown the E2F2 expression in RAW264.7. Western blot analysis of E2F2‐SLC7A5‐P70S6K signal and pathway influx of ^3^H‐leucine examination in RAW 264.7 showed similar results in BMDMs(Figure S3G,H, Supporting Information). Therefore, our findings reveal that E2F2 deficiency induces activated SLC7A5 expression and reprograms amino acid metabolism in macrophages.

### 2.4 E2F2 Deficiency Enhances Macrophage Proinflammatory Response by Promoting Glycolysis and Mitochondrial Dysfunction in a Leu‐mTORC1‐Dependent Manner

**Figure 4:**
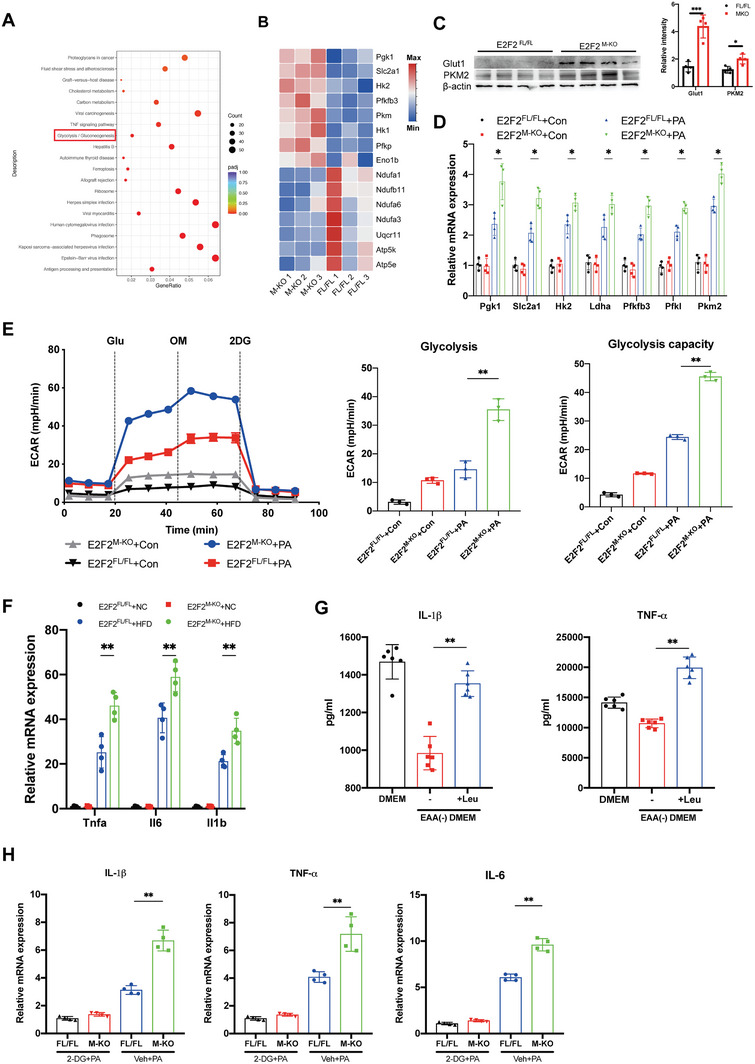
E2F2 deficiency enhances macrophage proinflammatory response by promoting glycolysis and mitochondrial dysfunction in a Leu‐mTORC1‐dependent manner. BMDMs were isolated from E2F2^M‐KO^ and E2F2^FL/FL^ mice and co‐cultured with primary hepatocytes with or without palmitic acid (PA) medium treated for 24 h. A,B) Heatmap (A) and GO (B) reveal the signature genes and up‐regulated signaling pathways related to glycolysis and mitochondrial function in BMDMs. n = 3/group. C) Western blot analysis of Glut1 and PKM2 in hepatic macrophages isolated from E2F2^M‐KO^ and E2F2^FL/FL^ mice fed with HFD. n = 6/group. D) The mRNA of glycolysis‐related genes (Pgk1, Slc2a1, Hk2, Ldha, Pfkfb3, Pfkl, and Pkm2) in BMDMs. n = 6/group. E) The glycolytic rate and capacities of BMDMs were measured by real‐time recording of extracellular acidification rates (ECAR) after injection of glucose (Glu), oligomycin (OM), and 2‐DG. n = 6/group. F) The mRNA level of Tnfa, il6 and il1b in BMDMs isolated from E2F2^M‐KO^ and E2F2^FL/FL^ mice. n = 6/group. G) The amount of cytokines in culture supernatant from BMDMs isolated from E2F2^M‐KO^ replenished with the indicated AAs. n = 6/group. H) mRNA expression of Tnf‐α, Il‐6, and Il‐1β in BMDMs isolated from E2F2^M‐KO^. n = 6/group. Data were presented as mean± SEM; *p ≤ 0.05, **p ≤ 0.01, ***p ≤ 0.001.

The stimulation of cellular glycolysis is linked to mTORC1, and glycolysis is necessary for macrophage activation.^[^ ^13^, ^30^, ^31^ ^]^ Consequently, we separated BMDMs from E2F2^M‐KO^ and E2F2^FL/FL^ mice and co‐cultured them in PA medium with primary hepatocytes obtained from WT mice. RNA sequencing was performed on BMDMs. KEGG analysis indicated that glycolysis and gluconeogenesis pathways were enriched in BMDMs from E2F2^M‐KO^ mice (**Figure** **4**A). The heatmap showed that the expression of genes related to glycolysis was up‐regulated and those related to oxidative phosphorylation (OXPHOS) were down‐regulated in BMDMs from E2F2^M‐KO^ mice (Figure 4B). Furthermore, we noticed that in hepatic macrophages separated from E2F2^M‐KO^ mice fed HFD, the expression of glycolysis‐related proteins (Glut1 and PKM2) increased (Figure 4C). The results of RNA‐sequence were confirmed by the mRNA levels of genes associated with glycolysis (Pgk1, Slc2a1, Hk2, Ldha, Pfkfb3, Pfkl, and Pkm2) (Figure 4D). Given the observed alterations in genes related to glycolysis and OXPHOS, we evaluated their corresponding indicators by measuring the extracellular acidification rate (ECAR) and oxygen consumption rate (OCR), respectively. E2F2 deficient macrophages have poorer mitochondrial respiration and higher glycolysis, as seen by the increased ECAR and decreased OCR observed in BMDMs from E2F2^M‐KO^ mice relative to BMDMs from E2F2^FL/FL^ mice. Notably, E2F2 deficiency significantly exacerbated proton leak which indicated mitochondrial damage in BMDMs from E2F2^M‐KO^ mice (Figure 4E and Figure S4A, Supporting Information).

In addition, mTORC1 is essential for controlling and affecting myeloid innate immune cells' effector responses. Therefore, in BMDMs from E2F2^M‐KO^ and E2F2^FL/FL^ mice co‐cultured with primary hepatocytes obtained from WT mice in PA media, we evaluated the mRNA levels of pro‐inflammatory cytokines. The results showed that E2F2 deficiency macrophages exhibit higher levels of inflammation (Figure 4F). The effect of mTORC1 activation, which is brought about by leucine influx mediated by SLC7A5, on the production of cytokines was then examined. In cultures depleted of EAAs, the levels of IL‐1β and TNF‐α in the culture supernatants were reduced. Nevertheless, Leu supplementation markedly elevated TNF‐α and IL‐1β levels (Figure 4G), and the glycolysis inhibitor 2‐deoxy‐D‐glucose (2‐DG) inhibited the rise in Tnfα, Il6, and Il1β expression seen in E2F2^M‐KO^ BMDMs as a result of the elevated glucose supply (Figure 4H).

mTORC1, a central regulator of autophagy, inhibits autophagy under nutrient sufficiency.^[^ ^32^ ^]^ Because of the finding of enhanced mTORC1 activation in macrophages from E2F2^M‐KO^ mice, we analyzed the autophagic flux in BMDMs from E2F2^M‐KO^ and E2F2^FL/FL^ mice co‐cultured with primary hepatocytes obtained from WT mice. The findings revealed a further rise in LC3‐II, but only in E2F2^FL/FL^ and not in E2F2^M‐KO^ BMDMs, suggesting that the latter had reduced autophagy (Figure S4B, Supporting Information). Meanwhile, DCFDA fluorescence and MitoSox staining found cell total and mitochondrial ROS were markedly accumulated in E2F2^M‐KO^ BMDMs(Figure S4C,D, Supporting Information). To further assess the functional significance of mTORC1 in regulating mitochondrial activity and macrophage activation, we employed Rapamycin, an mTOR inhibitor, along with LNAC, an antioxidant, to inhibit mTORC1 activation and scavenge ROS. As anticipated, the inhibition of mTORC1 activation and the scavenging of ROS led to a reduction in the activation of E2F2 deficiency macrophages(Figure S4E, Supporting Information). In conclusion, the E2F2 deficiency results in enhanced glycolysis and impaired mitochondrial function, and SLC7A5‐mediated AAs influx leading to macrophage activation in a Leu‐mTORC1‐dependent manner.

### 2.5 Knockdown of SLC7A5 in E2F2^M‐KO^ Mice Attenuates HFD Diet‐Induced Steatohepatitis

**Figure 5:**
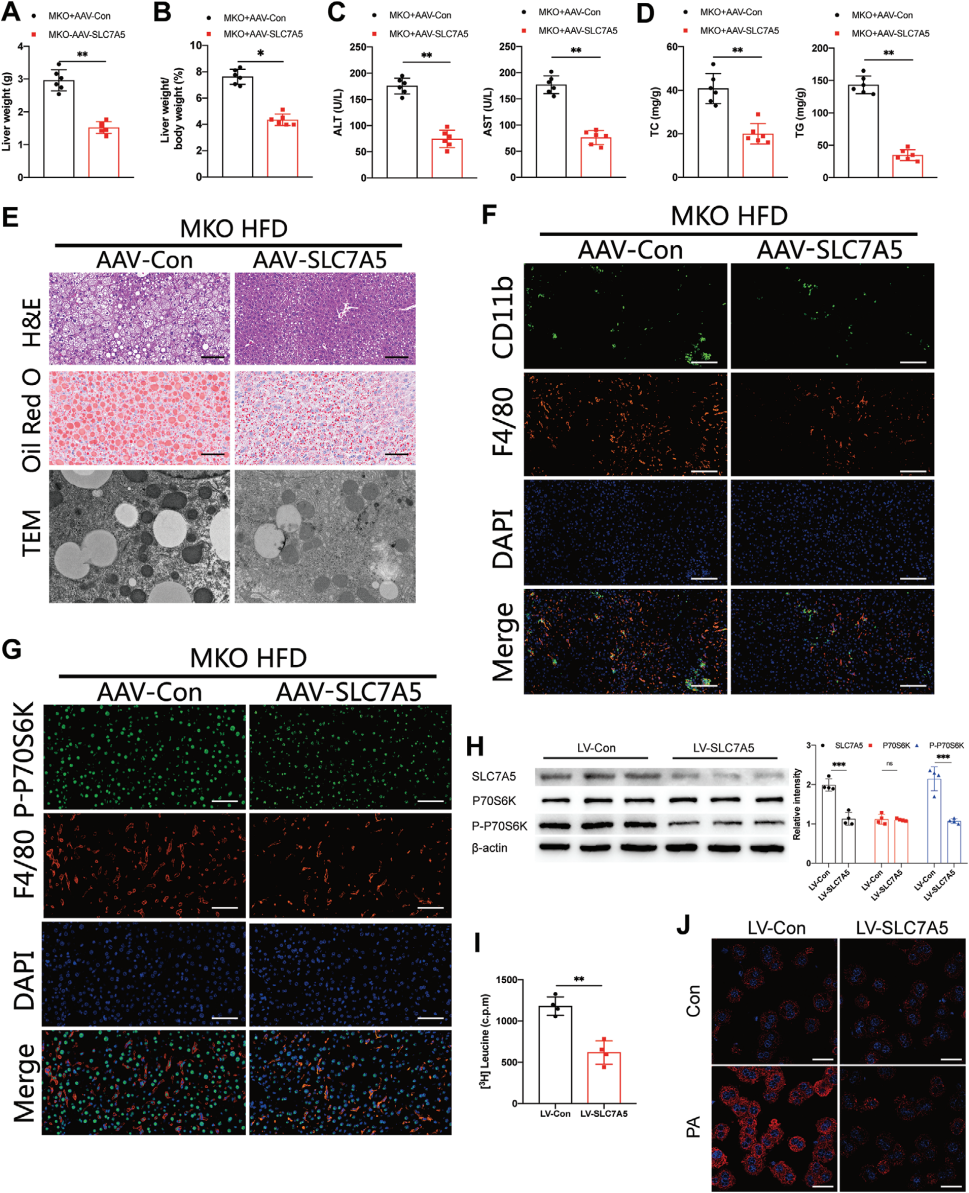
Knockdown of SLC7A5 in E2F2^M‐KO^ mice attenuates HFD diet‐induced steatohepatitis. A,B) Liver weights (A) and ratios of liver weight to body weight (B) of AAV‐Con or AAV‐SLC7A5 treated E2F2^M‐KO^ mice after HFD consumption. C) Serum ALT and AST concentrations in the indicated groups of mice. D) Hepatic TG and TC content of mice in the indicated groups. E–G) H&E, Oil Red O, TEM (E), CD11b and F4/80 (green and red) IF staining (F), P‐P70S6K and F4/80 (green and red)IF staining (G) in liver sections from AAV‐Con or AAV‐SLC7A5 treated E2F2^M‐KO^ mice fed with HFD. n = 6/group. H) Western blot analysis of SLC7A5, P70S6K, P‐P70S6K and β‐actin in BMDMs. I) Uptake of ^3^H‐leucine in BMDMs. BMDMs isolated from E2F2^M‐KO^ mice were treated with LV‐Con or LV‐SLC7A5, then co‐cultured with primary hepatocytes in palmitic acid (PA) medium for 24 h. J) representative Nile Red staining of primary hepatocytes co‐cultured with LV‐Con or LV‐SLC7A5 pre‐treated BMDMs isolated from E2F2^M‐KO^ mice with or without palmitic acid (PA) medium for 24 h. Data were presented as mean± SEM; Scale bars, 100µm; *p ≤ 0.05, **p ≤ 0.01, ***p ≤ 0.001.

To investigate the function of SLC7A5 in MASH in vivo and in vitro, AAV‐SLC7A5 and LV‐SLC7A5 were utilized to knockdown the expression of SLC7A5 in E2F2^M‐KO^ mice and in BMDMs from E2F2^M‐KO^ mice. LV‐E2F2‐OE was used to overexpress E2F2 in BMDMs from E2F2^FL/FL^ mice. In these AAV‐SLC7A5 treated E2F2^M‐KO^ mice, SLC7A5 expression was significantly knocked down in hepatic macrophages isolated from E2F2^M‐KO^ mice (Figure S5A, Supporting Information), although similar in hepatocytes (Figure S5B, Supporting Information). Analysis of HFD‐induced MASH indicated that E2F2^M‐KO^ mice with SLC7A5 knockdown exhibited decreased steatosis, reduced inflammation, lower liver weights, and diminished serum liver enzyme levels, along with decreased hepatic TG content(**Figure** **5**A–F). Furthermore, less expression of macrophage P‐P70S6K in liver sections of AAV‐SLC7A5‐treated E2F2^M‐KO^ mice was compared to AAV‐SLC7A5‐treated E2F2^M‐KO^ mice (Figure 5G).

We next co‐cultured E2F2^M‐KO^ BMDMs with primary hepatocytes obtained from WT mice in PA medium, blocking the SLC7A5 activation in the process. In agreement with the in vivo findings, the levels P‐P70S6K and pro‐inflammatory cytokines were significantly reduced in E2F2^M‐KO^ BMDMs treated with LV‐SLC7A5 (Figure 5H and Figure S5C–E, Supporting Information). The influx of ^3^H‐leucine was significantly decreased in E2F2 deficiency BMDMs subjected to LV‐SLC7A5 (Figure 5I). Quantitative real‐time PCR findings showed that in primary hepatocytes, suppression of SLC7A5 in E2F2^M‐KO^ BMDMs decreased the expression of lipogenic genes while boosting the expression of β‐oxidation‐related genes (Figure S5F, Supporting Information). The Nile Red staining of primary hepatocytes showed less lipid accumulation in LV‐SLC7A5 group compared to LV‐Con group (Figure 5J). Meanwhile, overexpression of E2F2 in E2F2^FL/FL^ BMDMs down‐regulates pro‐inflammatory cytokines release, lipid accumulation in primary hepatocytes, and expression of SLC7A5 and P‐P70SK6 (Figure S6A–E, Supporting Information). To elucidate the essential function of the SLC7A5‐Leu‐mTORC1 signaling pathway in the regulation of macrophage activation, we used MHY1485, a mTOR activator to treat E2F2^M‐KO^ BMDMs with LV‐SLC7A5 pretreatment. The MHY1485 group showed elevated levels of pro‐inflammatory cytokines (Figure S5G, Supporting Information). All of these findings pointed to the critical role that E2F2‐SLC7A5‐Leu‐mTORC1 signaling plays in controlling macrophage function during MASH development.

### 2.6 Macrophage E2F2 Deficiency Promotes Hepatocyte Lipid Accumulation and HSCs Activation

**Figure 6:**
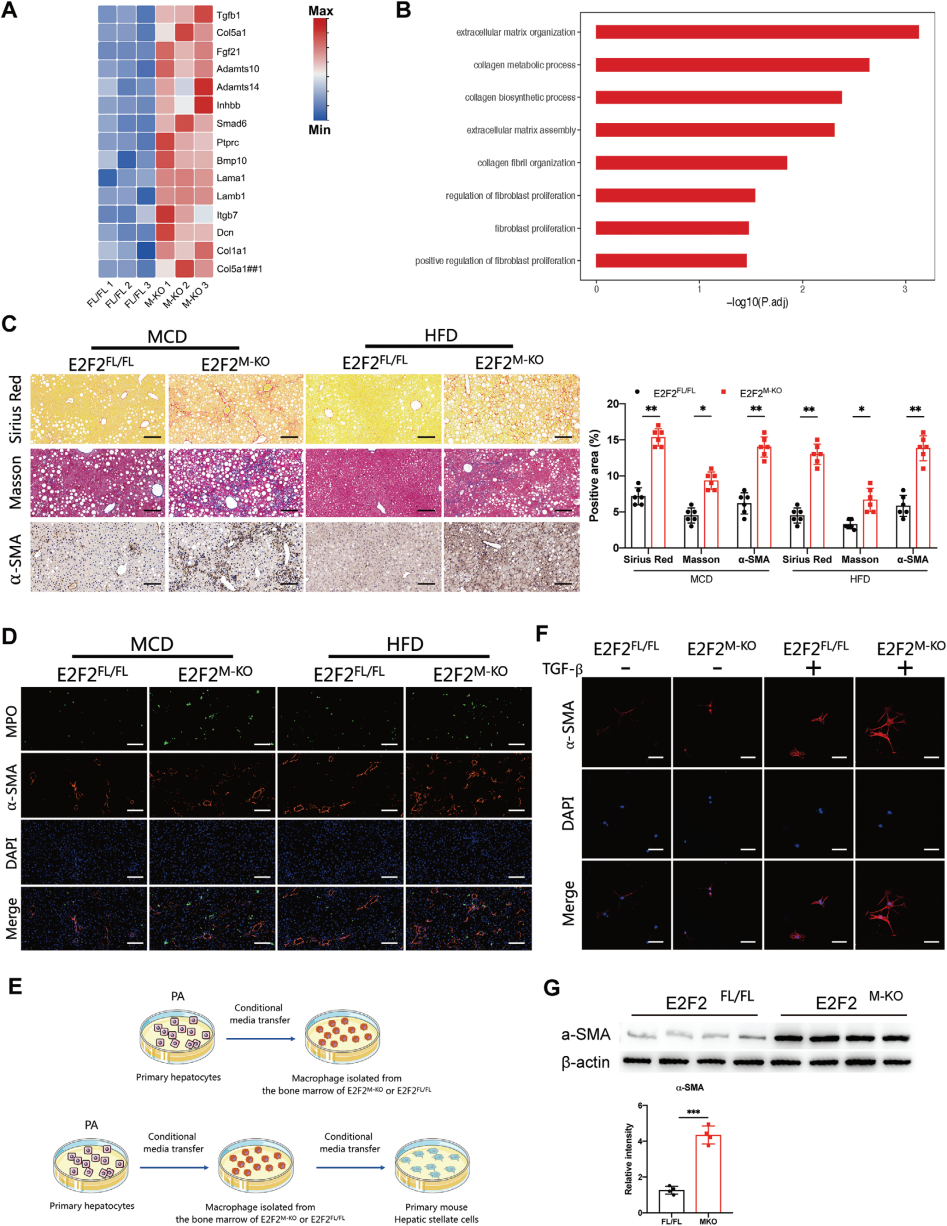
Macrophage E2F2 deficiency promotes hepatocyte lipid accumulation and hepatic stellate cell activation. A,B) Heatmap (A) and GO (B) reveal the signature genes and up‐regulated signaling pathways related to fibrosis in the liver samples of E2F2^M‐KO^ mice compared with E2F2^FL/FL^ mice. E2F2^M‐KO^ and E2F2^FL/FL^ mice were fed with HFD. (n = 3/group). C) Sirius Red, Masson, and α‐SMA IHC staining of liver sections from E2F2^M‐KO^ and E2F2^FL/FL^ mice fed with MCD or HFD. (n = 6/group). Scale bars, 100µm. D) Representative IF images showing MPO and α‐SMA (green and red) in liver sections from E2F2^M‐KO^ and E2F2^FL/FL^ mice fed with MCD or HFD. (n = 6/group). Scale bars, 100µm. E) Primary hepatocyte‐conditioned media was transferred to BMDMs for 48 h and then BMDM‐conditioned media was transferred to primary mouse hepatic stellate cells for 24 h. F,G) IF staining (F) and Western blot analysis (G) for α‐SMA in primary mouse hepatic stellate cells. (n = 6/group). Scale bars, 50µm. Data were presented as mean± SEM; *p ≤ 0.05, **p ≤ 0.01, ***p ≤ 0.001.

To explore the role of macrophage E2F2in fibrosis change in MASH progression, We reanalyzed the RNA sequencing data from liver samples mentioned above in Figure 2. The heatmap and GO analysis revealed that extracellular matrix receptor interaction and signature profibrotic genes were highly activated in the E2F2^M‐KO^ mice compared to E2F2^FL/FL^ mice (**Figure** **6**A,B). After 26 weeks of HFD or 6 weeks of MCD diet, we evaluated at the livers of E2F2^M‐KO^ and E2F2^FL/FL^ mice using Masson, α‐SMA, and Sirius Red staining. The findings showed that liver fibrosis severity increased in the mice on either diet, and this effect was exacerbated by the deficiency of macrophage E2F2 (Figure 6C). Furthermore, these findings were validated by α‐SMA and MPO immunofluorescence staining in liver slices (Figure 6D). Subsequently, we incubated HSCs with conditional medium (CM) from E2F2^M‐KO^ or E2F2^FL/FL^ BMDMs (Figure 6E). Increasing α‐SMA protein level revealed that the knockout of macrophage E2F2 significantly heightened HSCs activation (Figure 6F,G). Moreover, the knockdown of SLC7A5 expression in E2F2^M‐KO^ mice using AAV‐SLC7A5 reduced the extent of liver fibrosis in those mice subjected to HFD (Figure S7A,B, Supporting Information). Meanwhile, overexpression of E2F2 in E2F2^M‐KO^ BMDMs with LV‐E2F2‐OE suppresses HSCs activation (Figure S7C,D, Supporting Information). These data indicate that the depletion of macrophage E2F2 aggravates hepatic fibrosis in models of MASH and enhances the activation of HSCs.

### 2.7 Genetic or Pharmacological Activation of Macrophage Nrf2‐E2F2 Signaling Effectively Attenuates the MASH Progression

**Figure 7:**
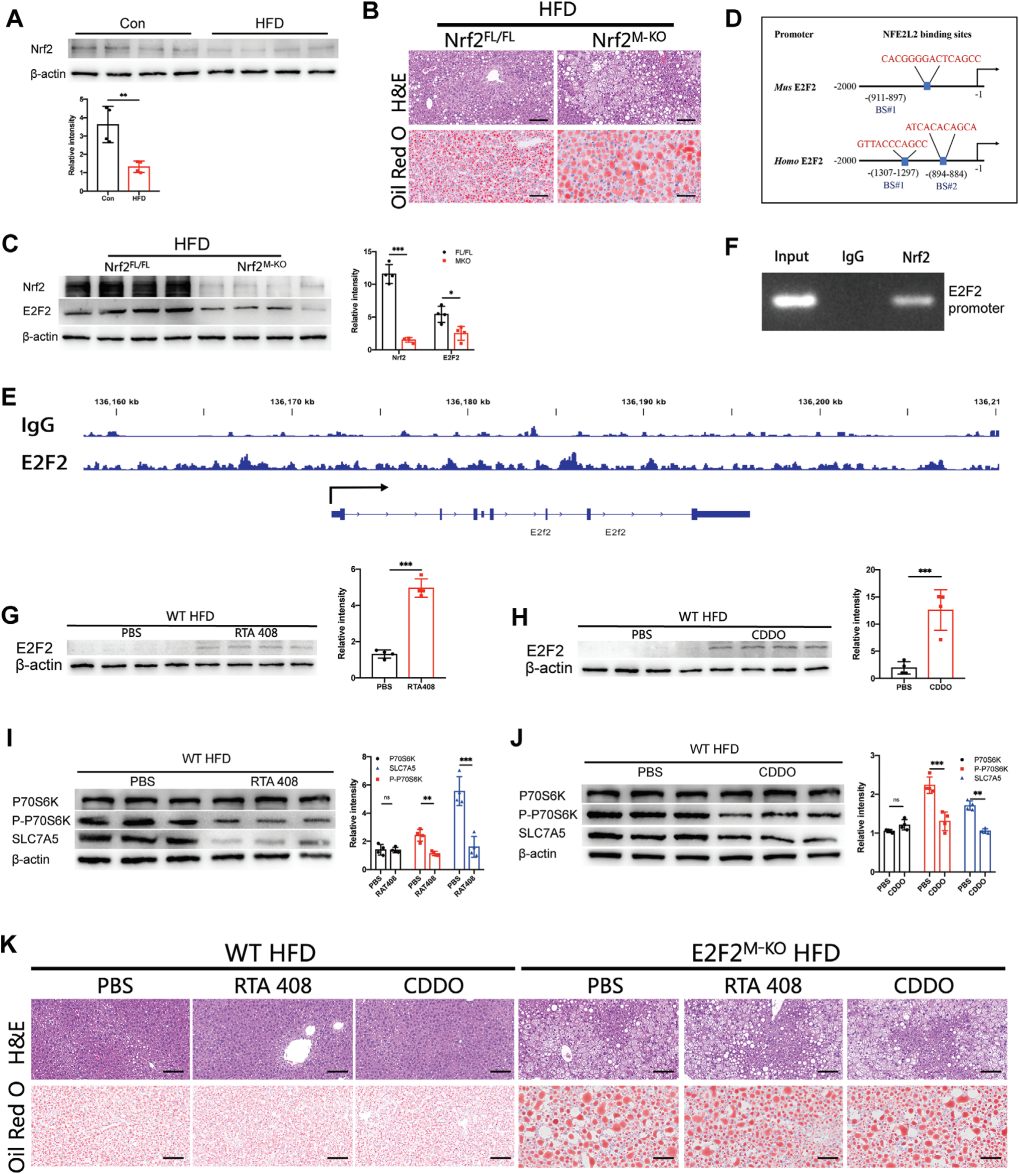
Genetic or pharmacological activation of macrophage Nrf2‐E2F2 signaling effectively attenuates the MASH progression. A) Nrf2 protein levels in hepatic macrophages isolated from WT mice livers. WT mice were fed the HFD, or control diet. B) Representative pictures in H&E and Oil Red O stained liver sections in Nrf2^M‐KO^ and Nrf2^FL/FL^ mice fed with HFD. C) Protein expression levels of Nrf2 and E2F2 in hepatic macrophages from Nrf2^M‐KO^ and Nrf2^FL/FL^ mice after HFD consumption. D) E2F2 promoter region targeted by the transcription factor, Nrf2. E) Representative Integrative Genomics Viewer (IGV) overview of CUT&Tag signals of E2F2 gene loci bound by Nrf2. F) ChIP‐PCR analysis of Nrf2 binding to the E2F2 promoter. G–J) Protein expression levels of E2F2, SLC7A5, P70S6K, P‐P70S6K and β‐actin in hepatic macrophages from WT mice after HFD consumption with PBS, RTA 408 (G and I) or CDDO (H and J) treatment. K) Representative pictures in H&E and Oil Red O stained liver sections in Nrf2^M‐KO^ and WT HFD‐fed mice with PBS, RTA 408 or CDDO. n = 6/group. Data were presented as mean± SEM; Scale bars, 100µm; *p ≤ 0.05, **p ≤ 0.01, ***p ≤ 0.001.

We subsequently investigated the factors that contribute to the downregulation of macrophage E2F2 in MASH. The earlier work found that Nrf2 suppresses apoptosis in an E2F2‐dependent way and increases antioxidant response to attenuate reactive oxygen species (ROS) and enhance cell development.^[^ ^23^ ^]^ In our earlier research, we discovered that Nrf2 is downregulated in MASH patients' hepatic macrophages^[^ ^33^ ^]^ and Nrf2 protein levels in these macrophages were lower in HFD‐fed mice's hepatic tissues than in mice given a control diet(**Figure** **7**A). In order to confirm that downregulated Nrf2 signaling in macrophages is responsible for the positive regulation of E2F2 expression, Nrf2^FL/FL^ and Nrf2^M‐KO^ mice were given the HFD diet for 26 weeks in order to induce MASH. MASH development was markedly severe in Nrf2^M‐KO^ mice (Figure 7B). Macrophages obtained from the liver tissues of both Nrf2^FL/FL^ and Nrf2^M‐KO^ mice were co‐cultured in PA media with primary hepatocytes extracted from Nrf2^FL/FL^ mice. Nile Red staining of the primary hepatocytes revealed that the deficiency of Nrf2 in macrophages led to increased lipid accumulation within the hepatocytes (Figure S8A, Supporting Information). The expression of E2F2 in macrophages was shown to be suppressed by Nrf2 loss, as demonstrated by Western blot examination of hepatic macrophages extracted from Nrf2^FL/FL^ and Nrf2^M‐KO^ mice fed an HFD diet (Figure 7C).

Subsequently, we utilized algorithms, such as the JASPAR database, to detect transcription factors that have interactions with E2F2. The information suggested that the promoter region of E2F2 and the transcription factor Nrf2 could interact (Figure 7D). Our results also showed unique peaks with enhanced genomic distribution patterns at the E2F2 promoter regions. Based on the CUT & Tag test results, this implies that Nrf2 and the E2F2 promoter region may interact(Figure 7E). In order to validate that Nrf2 is present in the E2F2 promoter area, primers were created to locate the Nrf2/TCF DNA‐binding site within the promoter using PCR (Figure 7F) Meanwhile, E2F2 and F4/80 IF analysis of liver sections indicated lower expression levels of E2F2 nuclear translocation in Nrf2^M‐KO^ mice than Nrf2^FL/FL^ mice fed with HFD (Figure S8B, Supporting Information). Furthermore, primary hepatocytes from Nrf2^FL/FL^ mice were co‐cultured with BMDMs obtained from Nrf2^FL/FL^ and Nrf2^M‐KO^ mice in PA media in order to confirm the preceding results in vitro. The IF staining and protein levels of E2F2 were decreased BMDMs isolated from Nrf2^M‐KO^ mice (Figure S8C,D, Supporting Information).

To deeply explore the connection between Nrf2 and E2F2, we overexpressed E2F2 with LV‐E2F2‐OE or LV‐Con in BMDMs from Nrf2^M‐KO^ mice and then co‐cultured with primary hepatocytes obtained from Nrf2^FL/FL^ mice. The Nile Red staining of primary hepatocytes showed less lipid accumulation in the LV‐E2F2‐OE group compared to the LV‐Con group (Figure S8E‐F). Next, we overexpressed Nrf2 with LV‐Nrf2 or LV‐Con in THP‐1 and then co‐cultured with HepG2. The results showed that the overexpression of Nrf2 in THP‐1 significantly alleviates the lipid accumulation in HepG2 (Figure S8G).

In order to investigate the translational possibility of Nrf2/E2F2 in macrophage and cause there is no E2F2 specific agonist, we established HFD‐induced mouse MASH model with Nrf2 specific agonists (RTA 408 and CDDO) or PBS treatment. Hepatic macrophages' protein level of E2F2 was increased by Nrf2‐specific agonists (Figure 7G,H), and inhibited the activation of SLC7A5‐P70S6K signaling pathway (Figure 7I,J). In WT mice, Nrf2‐specific agonists alleviate the development of experimental steatohepatitis, but not in E2F2^M‐KO^ mice, according to the results of H&E and Oil Red O (Figure 7K).

Lastly, we attempted to investigate the function of E2F2 in vivo by modulating human macrophage activation. Unfortunately, there are no drugs or inhibitors directly targeting E2F2 in the clinic currently. On the other hand, we observed that CDK4/6 inhibitors, whose mechanism of action is linked to decreased E2F target activity, have received FDA approval and are being used to treat breast cancer.^[^ ^34^, ^35^ ^]^ Thus, we gathered blood samples from fifteen breast cancer patients both before and after therapy with CDK4/6 inhibitors. After being separated, peripheral blood mononuclear cells (PBMCs) underwent further analysis. Using PBMCs, a Western blot revealed that CDK4/6 inhibitors enhanced SLC7A5 expression but reduced E2F2 expression (Figure S8H).

These results imply that there is a positive feedback link between E2F2 and Nrf2, and that macrophage Nrf2 down‐regulation lowers the expression of E2F2 in MASH. Clinical data indicated that CDK4/6 inhibitors may activate SLC7A5 by blocking E2F2 signal. Furthermore, E2F2/Nrf2 in macrophages is probably a potential clinical translational target for the treatment of MASH.

## 3 Discussion

The pathogenesis of MASH is associated with hepatocyte steatosis and necroinflammation, wherein liver macrophages play a crucial role in both processes.^[^ ^12^ ^]^ We have shown in this work that a decreased expression of macrophage E2F2 plays a role in the development of MASH. Our study revealed that E2F2 deficiency in murine macrophage results in significant lipid accumulation in hepatocytes, and inflammatory cell infiltration in mice liver which aggravated the development of different experimental steatohepatitis. Macrophage Nrf2 triggers transcription of E2F2 decreasing the lipid accumulation in hepatocytes. The expression of SLC7A5 is upregulated in E2F2 deficiency, and SLC7A5‐mediated AAs influx activates mTORC1 signaling, which in turn activates macrophages. Furthermore, the depletion of E2F2 enhanced glycolysis and impaired mitochondrial function by suppressing autophagy in macrophages. These findings indicate that E2F2 serves as a critical regulator in macrophages that are resistant to the development of MASH.

Growing evidence suggests that E2F family regulates mitochondrial function, carbohydrate metabolism, and participates in metabolic disorders.^[^ ^36^, ^37^ ^]^ Inactivation of E2F/Dp in fat body cells induces systemic metabolic alterations.^[^ ^38^ ^]^ Aberrant expression of E2F2 been strongly associated with poor outcomes in a range of diseases.^[^ ^39^, ^40^ ^]^ For instance, during MASLD‐related hepatocellular carcinoma, E2F2 is favorably linked with immune cell infiltration and creates a lipid‐rich tumor‐promoting milieu.^[^ ^25^ ^]^ SPINK1 facilitates self‐renewal, dedifferentiation, chemoresistance, and tumor initiation in HCC via a dysregulated regulatory axis involving CDK4/6 and E2F2.^[^ ^41^ ^]^ Moreover, E2F2 participates in regulating various immune cells’ functions including T cell^[^ ^26^ ^]^ and B cell.^[^ ^42^ ^]^ Nevertheless, further research is required to fully understand the precise role that E2F2 expression plays in the macrophage‐mediated immune response throughout the process of MASH. We have shown that during MASH, hepatic macrophages have down‐regulated levels of E2F2. Significantly, we examined that E2F2 deficiency regulated AAs metabolism to promote glycolysis and mitochondrial dysfunction, and in turn enhance macrophage proinflammatory response. Ultimately, this led to hepatocyte lipid buildup and HSC activation, which accelerated the development of steatohepatitis.

We previously reported that macrophage Nrf2 expression was diminished in MASH and macrophage Nrf2 regulated ROS‐mediated YAP signaling to slow down MASH progression.^[^ ^33^ ^]^ E2F2 promotes cells to generate antioxidants to counteract ROS‐induced cell death through activating Nrf2 signaling.^[^ ^23^ ^]^ Our results suggest that Nrf2^M‐KO^ mice showed a marked severe MASH development and macrophage Nrf2 up‐regulating the expression of E2F2 decreased the lipid accumulation in hepatocytes and suppressed the development of MASH. These findings support a positive feedback mechanism in which Nrf2 and E2F2 mutually enhance each other's expression, thereby inhibiting pro‐inflammatory immune activation of macrophages during the progression of MASH.

Mechanistically, we elucidate the molecular role of E2F2 in MASH and identify SLC7A5 as a target gene regulated by E2F2. SLC7A5, also known as l‐type amino acid transporter 1 (LAT1), cooperates with SLC3A2 to promote the absorption of large neutral amino acids by cells, such as tryptophan, phenylalanine, tyrosine, and leucine.^[^ ^43^ ^]^ Our results suggest that E2F2 was located on the promoter of SLC7A5 and inhibited the transcription of SLC7A5. Macrophage E2F2 knockout induced the upregulation of SLC7A5 expression. Amino acids are vital nutrients for immune cells, influencing their functionality. They are vital to metabolic reprogramming, which is necessary for immune cell activation.^[^ ^44^, ^45^ ^]^ LPS increases SLC7A5 expression and SLC7A5‐mediated AAs flux supports human pro‐inflammatory macrophage cytokine production.^[^ ^29^ ^]^ According to recent research, some amino acids—such as leucine and arginine—directly interact with certain sensing molecules to activate mTORC1.^[^ ^20^, ^21^ ^]^ Consequently, there has been a rise in interest in the modulatory effects of amino acids on this process, given the growing involvement of mTORC1 in glycolytic reprogramming inside monocytes and macrophages.^[^ ^16^, ^31^ ^]^ Additionally, pro‐inflammatory macrophages reduce OXPHOS and predominantly depend on glycolysis, the pentose phosphate pathway, and fatty acid synthesis for their energy needs.^[^ ^46^, ^47^ ^]^ In our work, we demonstrated that macrophage E2F2 depletion upregulated SLC7A5 expression leading to enhanced SLC7A5‐mediated AAs flux, and in turn, activated the mTORC1‐P‐P70S6K pathway in macrophage. Enhanced mTORC1 signaling led to an increase in glycolysis and inflammation. Meanwhile, the activation of mTORC1 inhibited autophagy and decreased the ability to scavenge ROS and mitochondrial ROS leading to impaired mitochondrial function in macrophages.

A vital prognostic factor for liver‐related outcomes and overall mortality in individuals with metabolic MASH is advanced liver fibrosis.^[^ ^48^ ^]^ Studies have indicated a strong association between the activation of HSCs and TGF‐β1 in the fibrogenesis process associated with the progression of MASH.^[^ ^49^ ^]^ Nonetheless, the precise mechanisms by which macrophages influence fibrogenesis in MASH remain unclear. TGF‐β1 expression was upregulated in this study as a result of macrophage E2F2 elimination, according to RNA sequencing analysis. Further investigations demonstrated that the absence of E2F2 in macrophages contributed to the activation of HSCs and the progression of liver fibrosis in MASH.

The ability to characterize cellular heterogeneity among hepatic macrophages in MASH is made possible by the extensive use of single‐cell RNA sequencing techniques.^[^ ^50^ ^]^ A unique subset of macrophages, defined by the expression of CD9 and TREM‐2, has been identified and referred to as “scar‐associated macrophages” (SAM). The location of SAMs in the fibrotic niche indicated that SAMs might promote liver fibrosis.^[^ ^51^ ^]^ Additionally, another distinct macrophage population known as “lipid‐associated macrophages” (LAMs) was observed periportally and near the bile ducts. It was discovered that LAMs grew in quantity and accumulated pericentrally in steatotic liver areas.^[^ ^52^ ^]^ We did not identify the alteration of E2F2 in each distinct macrophage subset in MASH, such as LAMs and SMAs and future research will aim to elucidate this change.

To summarize, our analysis has clarified the function of macrophage E2F2 in steatohepatitis and has demonstrated a negative correlation between the expression of E2F2 and the severity of liver disease in MASH patients. Macrophage Nrf2 up‐regulates E2F2 expression to resist the development of MASH. Macrophage E2F2 depletion increases the expression of SLC7A5 and in turn, SLC7A5‐mediated AAs influx enhances glycolysis in macrophages. Macrophage metabolic reprogramming increases pro‐inflammatory cytokine secretion, and HSC activation exacerbating the development of MASH. Furthermore, clinical research using CDK4/6 inhibitors not only confirmed our original MASH findings but also provided insight into the function and mode of macrophage E2F2 signaling in the context of anti‐tumor immunity. Consequently, our results provide a foundation for creating new treatment approaches meant to slow the advancement of MASH.

## 4 Experimental Section

### Human Liver and Blood Samples

This study included 31 individuals diagnosed with MASLD, 24 individuals with MASH, and 13 control participants with hemangioma who did not have MASLD or MASH, all of whom underwent partial liver resection at the First Affiliated Hospital of Nanjing Medical University (refer to Table S1, Supporting Information). Patients with MASH and those in the control group with peri‐tumor normal tissues provided liver biopsy samples. Two experienced pathologists, blinded to clinical information, independently evaluated all liver specimens based on the MASLD activity score (MAS). Furthermore, blood was drawn from 15 patients with breast cancer both before and after they received CDK4/6 inhibitor medication. The First Affiliated Hospital of Nanjing Medical University's Ethics Committee authorized all operations involving human samples, and each subject gave their informed permission.

### Animals and Treatments

This study utilized 6‐ to 8‐week‐old male mice with the following genotypes: wild‐type (WT), FloxP‐Nrf2 (Nrf2^FL/FL^), FloxP‐E2f2 (E2f2^FL/FL^), Lyz2‐Cre Nrf2 knockout (Nrf2^M‐KO^), and Lyz2‐Cre E2f2 knockout (E2f2^M‐KO^), all on a C57BL/6 background. The floxed alleles were bred to homozygosity to generate the Nrf2^FL/FL^ and E2f2^FL/FL^ mice. Nrf2^M‐KO^ and E2f2^M‐KO^ were produced by crossing Nrf2^FL/FL^ and E2f2^FL/FL^ with Lyz2‐Cre mice, maintaining the C57BL/6 genetic background. The mice were fed either a normal chow diet or HFD (Research Diets, Inc., New Brunswick, CA) for 26 weeks (*n* = 6–8 per group), or MCD (Research Diets, Inc., New Brunswick, CA) for 6 weeks (*n* = 6–8 per group). mice have unrestricted access to water and a 12 h light/dark cycle in housing. Food intake was recorded daily for one week, and body weight was measured each day. Following a fast at the conclusion of the trials, tissue and serum samples were obtained as previously mentioned, with a section of the liver set aside for RNA sequencing examination. The Affiliated Hospital of Nanjing Medical University's Institutional Animal Use and Animal Experimentation Ethics Committee established rules that were followed in all animal‐related procedures, which were carried out in a humane manner.

### Cell Culture and Treatment

Primary hepatocytes were obtained from liver intrahepatic macrophages from male Nrf2^FL/FL^, E2f2^FL/FL^, Nrf2^M‐KO^, and E2f2^M‐KO^ mice by a two‐stage collagenase perfusion method. For varying lengths of time, primary hepatocytes were subjected to treatments using palmitic acid (PA, 0.2 mm; Sigma–Aldrich) and bovine serum albumin (BSA, Sigma‐Aldrich), with BSA acting as the vehicle control. Using previously defined techniques,^[^ ^53^ ^]^ bone marrow cells were extracted from the femurs and tibias of male Nrf2^FL/FL^, E2f2^FL/FL^, Nrf2^M‐KO^, and E2f2^M‐KO^ mice. To facilitate additional experiments, BMDMs were re‐plated and cultivated overnight in fresh culture dishes.

For co‐culture studies, primary hepatocytes and macrophages were seeded on the co‐culture chamber (Corning Inc., NY, USA). The hepatocytes were examined for inflammatory response and fat formation after the co‐culture system was supplemented with palmitic acid media. BMDMs were incubated with hepatocytes conditional medium (hepatocytes‐CM) for 48 h and were subjected to RNA sequencing analysis and targeted amino acid metabolomics test. Primary mouse HSCs were incubated with BMDMs conditional medium (BMDMs‐CM).

### Adeno‐Associated Virus (AAV)‐Mediated Gene Transfer

For knockdown SLC7A5, the following Adeno‐associated virus serotype 2/8 (AAV2/8) under a macrophage‐special CD68 promoter was used in this study as previously reported^[^ ^54^ ^]^: AAV‐SLC7A5 (Shanghai Genechem). For the high‐fat diet‐induced MASH model, 6‐week‐old male E2F2^M‐KO^ mice were divided into two groups randomly. Then the mice were administered AAV‐SLC7A5 or AAV‐Con through the tail vein at 1×10^9^ IFU per 200 µL per mouse 2 weeks before all mice were fed with HFD. The adeno‐associated viruses were injected every 8 weeks. After being administered HFD for 26 weeks, all of the mice were sacrificed for additional examination. AAV‐Control was used as a negative control.

### Cell Metabolism Measurement

The XF96 analyzer (Agilent Technologies, Santa Clara, CA) measured the metabolism of BMDMs. BMDMs (1 × 10^5^ cells per well) were plated in a CellTak 96‐well plate. Various reagents were subsequently added to assess OCR using 1.5 µm oligomycin, 1.0 µm FCCP, 1.0 µm rotenone, and 1.8 µm antimycin A, as well as ECAR using 10 mm glucose, 2 µm oligomycin, and 50 mm 2‐deoxy‐d‐glucose. Glycolytic parameters were calculated using the XF glycolysis stress test report generator provided by the manufacturer. Specifically, glycolysis and glycolytic capacity were determined by subtracting ECAR after glucose treatment from ECAR before oligomycin treatment, and by subtracting ECAR after oligomycin from ECAR before 2‐DG treatment, respectively.

### Statistical Analyses

A two‐tailed Student's *t*‐test was used for comparisons between two groups, while a one‐way analysis of variance (ANOVA) followed by Bonferroni's post hoc test was employed for multiple group comparisons. Data are expressed as mean ± SD, with *p* ≤ 0.05 considered statistically significant. Statistical analysis was conducted using GraphPad Prism Version 7.0.

Details on other materials and methods are provided in the Supporting Information.

### Ethics Approval

The study design and sample collection protocols received approval from the Ethical Board of the First Affiliated Hospital of Nanjing Medical University (Serial number: 2022‐SRFA‐392). All animals were treated humanely, and all procedures complied with the relevant legal and ethical requirements, adhering to the protocols (number NMU08‐092) approved by the Institutional Animal Care and Use Committee of Nanjing Medical University.

## Conflicts of interest

The authors declare no conflict of interest.

## Author Contributions

Z.L., H.W., Y.L., and M.L. contributed equally to this work and are co‐first authors. Z.L., H.W., Y.L., M.L., M.W., J.Z., and Q.B. performed the experiments and analyzed the data. L.L. and H.Z. designed the experiments and drafted the manuscript.

## Supporting information



Supporting Information

## Data Availability

Research data are not shared.
