# Correction to “[E2F2 Reprograms Macrophage Function By Modulating Material and Energy Metabolism in the Progression of Metabolic Dysfunction‐Associated Steatohepatitis]”

**DOI:** 10.1002/advs.202502901

**Published:** 2025-03-07

[Liu Z, Wang H, Liang Y, Liu M, Huang Q, Wang M, Zhou J, Bu Q, Zhou H, Lu L. E2F2 Reprograms Macrophage Function By Modulating Material and Energy Metabolism in the Progression of Metabolic Dysfunction‐Associated Steatohepatitis. *Adv. Sci. (Weinh)*. 2024 Dec;11(48):e2410880.]

https://doi.org/10.1002/advs.202410880

[Specifically, the representative P70S6K Western blot image for hepatic macrophages from WT mice after HFD consumption with PBS or RTA408 treatment in Figure 7I was mistakenly used in the place of hepatic macrophages from WT mice after HFD consumption with PBS or CDDO treatment in Figure 7J.]


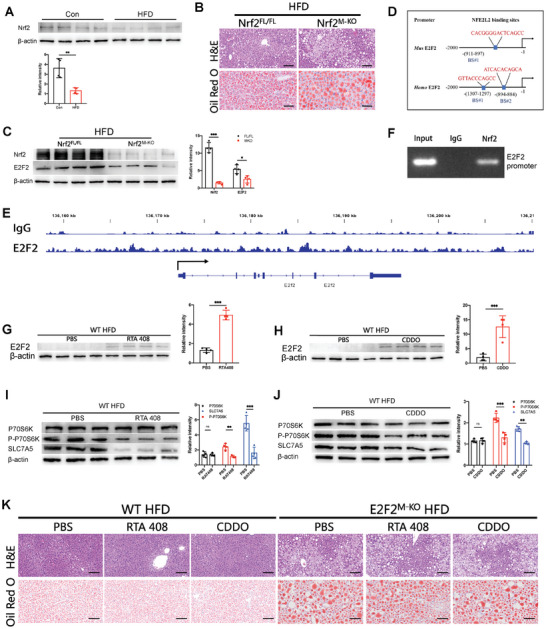


We apologize for this error.

