# Supplementary material for: E2F2 Reprograms Macrophage Function By Modulating Material and Energy Metabolism in the Progression of Metabolic Dysfunction‐Associated Steatohepatitis
Source: Adv Sci (Weinh). 2024 Oct 28;11(48):2410880. doi: 10.1002/advs.202410880 (PMC11672278; doi:10.1002/advs.202410880)
Supplement: Supplementary file 1 — Supporting Information [file ADVS-11-2410880-s001.docx]

**Materials and methods**

**Histological examination**

Hematoxylin and eosin (H&E)-stained liver tissue slices were evaluated by two pathologists who were blinded to their assessments. The evaluation determined the presence of MASH, guided by the MASLD activity score (MAS score). To do Oil Red O staining, 10 μm frozen liver sections were rinsed with 60% isopropanol, then treated for 15 minutes with Oil Red O solution (Sigma-Aldrich). Finally, an extra rinse and nuclear staining with hematoxylin were performed.

**CUT & Tag assay**

The DNA-binding profiles of Nrf2 and E2f2 were assessed by using the Hyperactive In-Situ ChIP Library Prep Kit (pG-Tn5) and Hyperactive Universal CUT &Tag Assay Kit for Illumina (Vazyme Biotech Co., Ltd#TD901-01 and TD903-01) according to the manufactures’ instructions.

**Luciferase reporter assay**

Luciferase reporter assays were conducted following the manufacturer's instructions. In short, 24-well plates were seeded with BMDMs, which were then transfected with 1 μg of mutant or shortened SLC7A5 and E2F2 promoter luciferase reporters. For normalization, 0.025 μg of pRL-TK was added. Following 48 hours of transfection, the cells were collected in lysis buffer, and the Dual-Luciferase Reporter Assay System (Promega, Madison, WI) was utilized to assess luciferase activity in accordance with the manufacturer's instructions.

**Immunohistochemical and immunofluorescence staining**

Immunohistochemistry for α-SMA in mouse liver tissue was performed using an anti-α-SMA antibody (#19245S, Cell Signal Technology). The secondary antibody was goat anti-rabbit IgG that had been biotinylated (Vector, CA, USA). It was then incubated with an immunoperoxidase reagent (ABC Kit, Vector) according to the manufacturer's instructions. CD68 in human liver tissues was detected by immunofluorescence with anti-rabbit CD68 mAb (ab955, Abcam), CD11b, MPO, $\alpha$-SMA, Nrf2, E2F2, SLC7A5, and P-P70S6K in human liver tissues, mice liver tissues, and cells was detected by immunofluorescence with anti-rabbit CD11b mAb (ab13357, Abcam), anti-rabbit MPO mAb (ab208670, Abcam), anti-rabbit $\alpha$-SMA mAb (#19245S, Cell Signal Technology), anti-rabbit Nrf2 mAb (16396-1-AP, Proteintech), anti-rabbit E2F2 mAb (AF4100, Affinity), anti-rabbit SLC7A5 mAb (DF8065, Affinity), and anti-rabbit P-P70S6K mAb (AF3228, Affinity), Anti-rabbit F4/80 mAb (ab300421, Abcam) was used to identify F4/80 in mouse liver tissues by immunofluorescence. This was followed by an incubation with secondary goat anti-rabbit IgG (ab150088, Abcam) or goat anti-mouse IgG (ab150117, Abcam). As directed by the manufacturer, mitochondrial reactive oxygen species (ROS) were measured using the MitoSOX Red Mitochondrial Superoxide Indicator (40778ES50, Yeasen). For nuclear labeling, DAPI and Hoechst 33342 (Invitrogen, H3570) were utilized. A Zeiss LSM880 confocal microscopy equipment was used to photograph the stained sections, and Zen software was used for analysis.

**Cell transfection**

The SLC7A5-shRNA lentiviruses (LV-SLC7A5, Shanghai Genechem), E2F2-shRNA lentiviruses (LV-E2F2, Shanghai Genechem) and E2F2 lentiviruses (LV-E2F2-OE, Shanghai Genechem) were used to knockdown SLC7A5, E2F2 and overexpress E2F2 in BMDMs. 1 × 10^6^ lentivirus transducing units were used to infect target cells (2 × 10^5^) for 72 hours at 37°C with 4 mg/ml polybrene present. Moreover, vectors denoted as lentiviral plasmid negative control (LV-Con) were produced.

**^3^H-Amino Acid Uptake Assay**

Following the removal of the culture medium, the cells were initially incubated in HBSS for ten minutes. The ³H-leucine (Perkin Elmer, Waltham, MA, USA) uptake assay commenced with the addition of HBSS containing 0.5-1 μCi, in which the cells were incubated for 15 minutes. Following three washes with ice-cold HBSS, the cells were detached using 1 M NaOH. Lastly, a beta scintillation counter (MicroBeta®; Perkin Elmer) was used to measure radioactivity.

**RNA extraction and quantitative polymerase chain reaction (qPCR)**

Total RNA was extracted from liver tissues or cells using TRIzol reagent (Invitrogen) and subsequently reverse transcribed into cDNA using a high-capacity cDNA reverse transcription kit (Roche) following the manufacturer’s instructions. mRNA levels were quantified using quantitative PCR with SYBR Green (Roche), and all quantitative real-time PCR experiments were conducted in triplicate. The results were normalized to β-actin for consistency.

**Protein extraction and western blot**

Proteins were extracted from liver tissue or cells using ice-cold lysis buffer composed of 0.5% sodium deoxycholate, 1% Triton X-100, 10% glycerol, 0.1% SDS, 137 mM sodium chloride, 20 mM Tris, adjusted to pH 7.4. A total of 20 μg of protein per sample was subjected to 10% SDS-PAGE electrophoresis and subsequently transferred to a PVDF nitrocellulose membrane (Bio-Rad). Anti-E2F2 (16396-1-AP, Proteintech), anti-Nrf2 (#12721S, Cell Signal Technology), anti-SLC7A5 (DF8065, Affinity), anti-P-P70S6K (AF3228, Affinity), anti-$\alpha$-SMA mAb (#19245S, Cell Signal Technology), anti-LC3B (#43566S, Cell Signal Technology), anti-p62 (#39749S, Cell Signal Technology) and $\beta$-actin (#3700S, Cell Signal Technology) were used in western blot analysis and $\beta$-actin was served as the control.

**Enzyme-linked immune absorbance assay (ELISA)**

Serum and medium cytokines, including TNF-α, IL-6, and IL-1β, were quantified according to the manufacturer's protocols using ELISA kits (Thermo Fisher Scientific, MA, USA).

**Transmission electron microscopy (TEM)**

Transmission electron microscopy (TEM) of primary hepatocytes and liver tissue was conducted following the manufacturer’s instructions. The sections were then stained with 0.3% lead citrate and imaged using an electron microscope (HITACHI, Tokyo, Japan).
